# Identification of Eight Different Isoforms of the Glucocorticoid Receptor in Guinea Pig Placenta: Relationship to Preterm Delivery, Sex and Betamethasone Exposure

**DOI:** 10.1371/journal.pone.0148226

**Published:** 2016-02-03

**Authors:** Zarqa Saif, Rebecca M. Dyson, Hannah K. Palliser, Ian M. R. Wright, Nick Lu, Vicki L. Clifton

**Affiliations:** 1 Mater Medical Research Institute, University of Queensland, Brisbane, Qld, Australia; 2 Robinson Research Institute, School of Paediatrics and Reproductive Health, University of Adelaide, Adelaide, SA, Australia; 3 Mothers and Babies Research Centre; Hunter Medical Research Institute, New Lambton Heights, NSW, 2305, Australia; and Faculty of Health, University of Newcastle, Callaghan, NSW, 2308, Australia; 4 Graduate School of Medicine, University of Wollongong and Illawarra Health and Medical Research Institute, Wollongong, NSW, 2522, Australia; 5 Division of Allergy-Immunology, Department of Medicine, Northwestern University, Chicago, IL, United States of America; University of Missouri, UNITED STATES

## Abstract

The placental glucocorticoid receptor (GR) is central to glucocorticoid signalling and for mediating steroid effects on pathways associated with fetal growth and lung maturation but the GR has not been examined in the guinea pig placenta even though this animal is regularly used as a model of preterm birth and excess glucocorticoid exposure. Guinea pig dams received subcutaneous injections of either vehicle or betamethasone at 24 and 12 hours prior to preterm or term caesarean-section delivery. At delivery pup and organ weights were recorded. Placentae were dissected, weighed and analysed using Western blot to examine GR isoform expression in nuclear and cytoplasmic extracts. A comparative examination of the guinea pig GR gene identified it is capable of producing seven of the eight translational GR isoforms which include GRα-A, C1, C2, C3, D1, D2, and D3. GRα-B is not produced in the Guinea Pig. Total GR antibody identified 10 specific bands from term (n = 29) and preterm pregnancies (n = 27). Known isoforms included GRγ, GRα A, GRβ, GRP, GRA and GRα D1-3. There were sex and gestational age differences in placental GR isoform expression. Placental GRα A was detected in the cytoplasm of all groups but was significantly increased in the cytoplasm and nucleus of preterm males and females exposed to betamethasone and untreated term males (KW-ANOVA, P = 0.0001, P = 0.001). Cytoplasmic expression of GRβ was increased in female preterm placentae and preterm and term male placentae exposed to betamethasone (P = 0.01). Nuclear expression of GRβ was increased in all placentae exposed to betamethasone (P = 0.0001). GRα D2 and GRα D3 were increased in male preterm placentae when exposed to betamethasone (P = 0.01, P = 0.02). The current data suggests the sex-specific placental response to maternal betamethasone may be dependent on the expression of a combination of GR isoforms.

## Introduction

Preterm delivery (gestation <37 completed weeks) is a major cause of neonatal mortality and morbidity. Betamethasone treatment for women at risk of preterm delivery is considered an essential intervention for fetal lung maturation and neonatal survival especially in infants delivered less than 34 weeks of gestation. However the long term effects of excess glucocorticoid exposure *in utero* continue to be examined with evidence from animal and human studies suggesting there are effects on neurodevelopmental, cardiovascular and metabolic pathways. This however remains an area of controversy as follow up studies in adults exposed to exogenous glucocorticoids *in utero* and who delivered preterm would indicate no short [1] or long term effect of this exposure on health [2–5] with only minor differences in insulin sensitivity and adiposity by 35 years of age [6,7].

Sheep studies have identified betamethasone was more effective at inducing fetal lung maturation when administered to the mother than when administered directly to the fetus[8]. However maternal betamethasone administration also resulted in a fetal growth reduction [9] suggesting exogenous glucocorticoid administration may exert some of its effects on the fetus via changes in placental function [10]. Subsequent studies identified increased placental apoptosis [11] and decreased circulating insulin-like growth factor concentrations [12] following maternal betamethasone exposure which in turn may influence placental nutrient transport and fetal growth. Similar changes in guinea pig fetal growth with maternal betamethasone exposure have been reported [13] but placental function in relation to the impact of glucocorticoids has not been studied in great depth in this particular species.

The placental glucocorticoid receptor (GR) is central to glucocorticoid signalling and mediating steroid effects on fetal growth and lung maturation but GR has not been examined in the guinea pig placenta even though this animal is regularly used as a model of preterm birth and excess glucocorticoid exposure. GRα A, the bioactive isoform of the receptor, is normally studied in detail when examining the impact of glucocorticoids. However numerous studies have reported there are several translational and splice variant isoforms of the GR that play an active role in glucocorticoid biology. The GR is a ubiquitously expressed nuclear receptor comprised of 9 exons. Exon 1 of the GR gene has a 5’ untranslated region which can be spliced into 9 different promoter variants[14] that function in a tissue specific manner to regulate GR protein expression. Exons 2–9 can generate various isoforms of GR through alternative splicing [15–17] or through alternative initiation of translation [17,18] resulting in the expression of GRα, GRβ, GRγ, GR-A and GR-P proteins. GRα can be expressed as eight different translational isoforms that originate from GRα mRNA through multiple start codons present on exon 2. These include GRα-A (94 kD) and GRα-B (91 kDa), GRα C1-C3 (82–84 kDa) and GRα D1-D3 (53–56 kDa). It is suggested that the various translational isoforms of GRα function in a tissue specific manner and have the ability to translocate into the nucleus to regulate transcriptional activities [17]. The splice variants such as GRβ, inhibit activation of GRα through a dominant negative mechanism. Splice variants GRγ, GR-A and GR-P have low transactivation activities [19,20]. Recently it was identified in the human placenta there were 12 different molecular weight (MW) proteins specific for the GR antibody and eight of these MWs were equivalent to known isoforms including GRα A (94kDa), GRβ (91 kDA), GRα C (81 kDa) GRP (74kDa) GRA (65 kDa), GRα D1-3 (50–55 kDa) [21]. Given the guinea pig has a haemochorial placenta similar to that of the human, the current study was designed to examine guinea pig placental GR and determine if there are other isoforms of the GR present. GR expression was examined in guinea pig placentae in relation to gestational age at delivery, fetal sex and betamethasone exposure.

## Methods

### Animals

All procedures were approved by the University of Newcastle Animal Care and Ethics Committee and were performed in accordance with the Australian Code of Practice for the Care and Use of Animals for Scientific Purposes. The Research Support Unit of the University of Newcastle supplied time-mated, pregnant outbred, tricolour guinea pigs at 21d gestational age (GA). Normal gestation in this population is 71d [22]. Dams were handled regularly by research staff from early in the pregnancy to reduce near term animal stress and subsequent cortisol release as a confounding factor.

Guinea pig dams received subcutaneous injections of either vehicle (175μL/kg, 0.9% sodium chloride) or betamethasone (Celestone Chronodose, 1mg/kg, Schering-Plough, Sydney, Australia) 24 and 12 hours prior to preterm (62±1d GA) or term (68/69d GA) caesarean-section delivery. The four resulting fetal groups were defined as control (delivery at 69d GA + vehicle treatment), term betamethasone exposure (delivery at 69d GA + betamethasone treatment), preterm (delivery at 62d GA + vehicle treatment) and preterm betamethasone exposure (delivery at 62d GA + betamethasone treatment). The treatment groups were further subdivided by sex. There were 6–8 dams per group, with only one dam being represented by more than one fetus per sex.

### Tissue Collection

The dams were anesthetized for surgical delivery by administering 4% isoflurane in medical grade oxygen using chamber and mask inhalation for 15 min before delivery. At delivery, the uterus was exposed by means of an incision made down the midline of the ventral abdomen and removed from the abdominal cavity maintaining blood supply. Dams and fetuses were euthanased by intracardiac administration of sodium pentobarbitone (200mg/kg, Lethabarb, Virbac, Milperra, Australia) following cardiac puncture and blood collection.

Pup and organ weights were recorded. Placentae were dissected, weighed and the right half was snap frozen and stored at -80°C until analysis and the left side was placed in fixative solution.

### Western blot

#### Cellular fraction preparation from placental tissue

Placental tissue was homogenised in complete cytosolic fractionation buffer [23] containing complete protease inhibitor cocktail. Lysates were spun at 8000 rpm for 5 min. Supernatants were kept for the cytosolic fraction. Nuclear fraction buffer with complete protease inhibitor cocktail was added to the pellet. Nuclear lysates were rotated at 4C° for 30 min, sonicated twice for 10 sec at 30% amplitude using an ultrasonic processor VCX 130 (Sonics, USA) and spun at full speed for 8 min to remove debris. Supernatants were stored at -80°C. Protein concentrations for each fraction were measured using Bradford assay.

#### Visualisation of target proteins

Cytosolic and nuclear protein fractions (60 μg) were electrophoresed on 3–8% Tris-acetate precast gels (Invitrogen, Life technologies, Carlsbad, California, USA) as previously described [24]. Blots were incubated with rabbit anti human-GR total (1:1500) (Bethyl Laboratories, Montgomery, TX, USA, S1 Table) antibody targeted to residue 150–200 of the GR receptor and expected to cross react with other animal species including bovine, ovine, equine and primate. The appropriate secondary antibody (goat anti-rabbit, goat anti-mouse or donkey anti-goat 1:2500) was applied for 1 hr. Membranes were subsequently probed with anti-β actin (1:2500, Abcam laboratories, UK, S1 Table) and anti-lamin A/C (1:1500, Santa Cruz Biotechnology, Santa Cruz, California, USA, S1 Table) antibodies as loading controls for cytoplasmic and nuclear fractions, respectively. The densitometric analysis was carried out using G:BOX Chemi Gel Imaging Systems (SYNGENE) to quantify the expression levels of different GR isoforms relative to β actin. Peptide competition with anti GR total antibody (1μg/1.5ml) incubated with 1X (1μg) and 2X (2μg) concentration of the control peptide (Bethyl Laboratories, USA) was performed as a specificity control.

#### GR Sequence Analysis

Human (NP_000167.1), monkey (XP_001097126.1) rat (NP_036708.2), mouse (NP_032199.3), and guinea pig (NP_001166458.1) GR sequences were obtained from the National Center for Biotechnology Information. The alignment of the sequences was performed using Sequence Analysis (http://informagen.com/SA/).

#### Statistical analysis

Statistical analysis was performed using the Statistical Package for the Social Sciences (SPSS v 19). GR data was not normally distributed so non-parametric tests were used which included Mann-Whitney tests and Kruskal Wallis ANOVA. The alpha level was set at 0.05. GR data is expressed as median and interquartile range. For normally distributed data, the data was expressed as the mean with the standard error of the mean (SE) and analysed using the Students t test and ANOVA where appropriate.

## Results

There were significant differences between term and preterm pregnancies in relation to maternal age at mating (ANOVA, P = 0.0001), weight gain from mid-gestation to delivery (P = 0.002), gravidity (P = 0.0001), birthweight (P = 0.0001) and gestational age at delivery (P = 0.0001) (Table 1). Post hoc tests identified maternal age at mating was significantly less in the male preterm group, female and male term groups exposed to betamethasone. Weight gain from mid-gestation was less in female and male term groups exposed to betamethasone. Gravidity was slightly less in the male preterm control group. Birthweight was significantly reduced in male preterm group exposed to betamethasone (Bonferroni post hoc test, P = 0.018). Placental weight was not significantly different between preterm or term pregnancies and was not affected by betamethasone treatment or sex.

**Table 1:** Maternal and Neonatal Characteristics.

Maternal and Neonatal Characteristics									
	Control Preterm		Control Term		Steroid Preterm		Steroid Term		P Value
	Female	Male	Female	Male	Female	Male	Female	Male	
n	6	7	6	8	6	8	8	7	
**Age at mating (months)**	8.83	8.75	11.83	13	8.83	6	6.75	5.85	0.000
*SE*	*1*.*7*	*1*.*25*	*1*.*42*	*1*.*29*	*3*.*4–7*.*9*	*0*.*91*	*1*.*14*	*0*.*91*	
**Maternal Weight (kg)**	1.11	1.13	1.32	1.3	1.1	0.88	2.48	1.29	
*SE*	*0*.*04*	*0*.*03*	*0*.*04*	*0*.*04*	*0*.*04*	*0*.*17*	*1*.*33*	*0*.*22*	0.520
**Weight gain from mid gestation (%)**	18	19.5	34	36.25	19.6	31.8	44.6	42.7	0.000
**Gravidity**	4	4.4	3	3.12	3.17	2.5	2.88	2.42	0.000
*SE*	*0*.*51*	*0*.*29*	*0*.*44*	*0*.*29*	*0*.*31*	*0*.*18*	*0*.*35*	*0*.*29*	
**Steroid dose (mg/kg)**	0	0	0	0	200.75	206	193.3	194.4	0.990
*SE*	*0*	*0*	*0*	*0*	*4*.*75*	*3*.*62*	*5*.*1*	*9*.*97*	
**Gestational age at delivery (days)**	62.17	62.29	68.17	68.13	62	61.75	68.5	68.57	0.000
*SE*	*0*.*17*	*0*.*18*	*0*.*17*	*0*.*13*	*0*.*25*	*0*.*25*	*0*.*2*	*0*.*19*	
**Birthweight (g)**	64.83	61.81	83.33	80.22	62.35	56.52	85	91.92	0.000
*SE*	*3*.*14*	*3*.*33*	*6*.*26*	*6*.*99*	*4*.*5*	*1*.*28*	*5*.*5*	*4*.*67*	
**Placental Weight (g)**	4.05	4.07	4.53	4.27	3.97	4.56	4.29	3.58	0.540
*SE*	*0*.*37*	*0*.*26*	*0*.*54*	*0*.*5*	*0*.*25*	*0*.*31*	*0*.*26*	*0*.*23*	
**Litter size**	3.33	3.14	5.33	4.63	3.33	4.38	3.25	3.71	0.100
*SE*	*0*.*49*	*0*.*34*	*0*.*99*	*0*.*67*	*0*.*76*	*0*.*49*	*0*.*37*	*0*.*28*	

Data is expressed as mean and standard error of the mean (SE).

### Guinea Pig GR Gene

**Fig 1:**
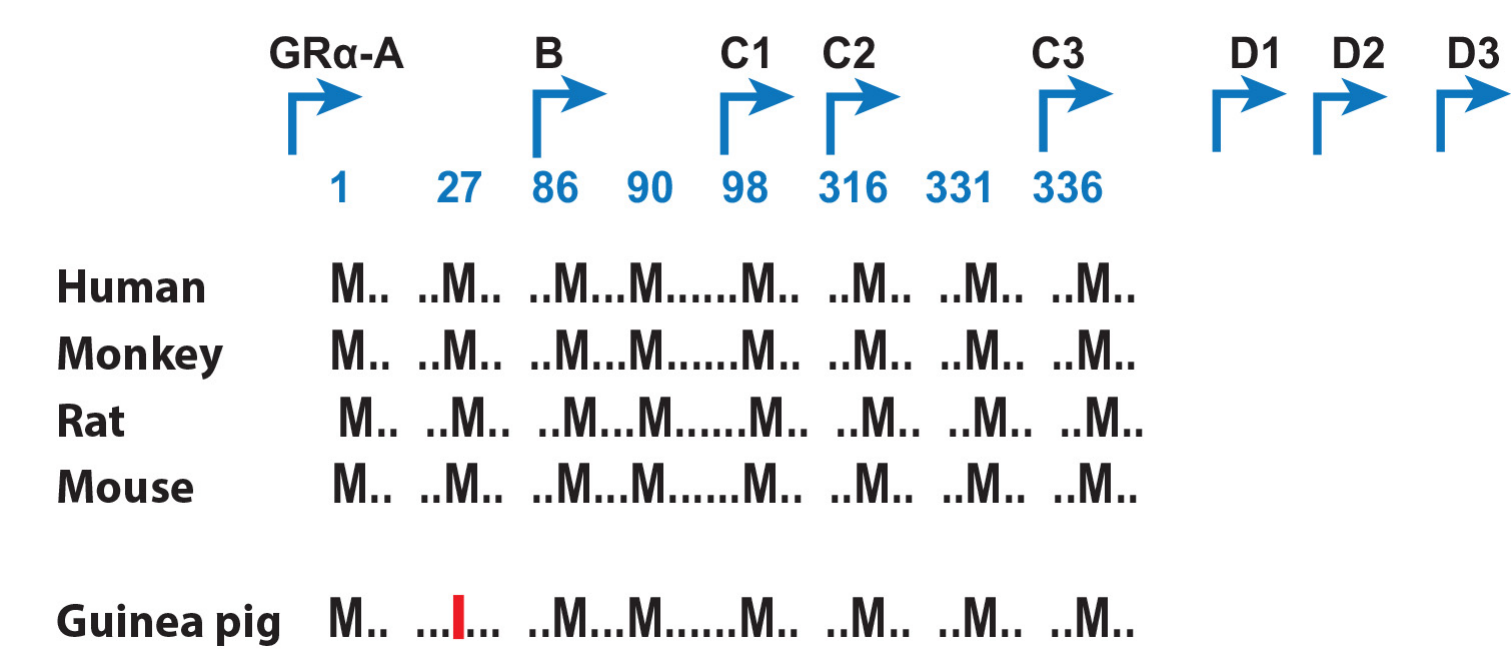
Comparison of Exon 2 start codons for the NCR31 gene in the guinea pig, Human, Monkey, Rat and Mouse. Start codons are positioned at methionine residues 1, 27, 86, 90, 98, 316, 331, 336 which result in the translation of GRα-A, B, C1, C2, C3, D1, D2, and D3. GRα-B is not translated in the guinea pig due to the presence of isoleucine at position 27. Alternative start codon (M, AUG) positions of human GR are indicated in the diagram. M27 in guinea pig GR was replaced by an isoleucine (I).

A comparative examination of the guinea pig GR gene identified that it is capable of producing seven of the eight translational GR isoforms which include GRα-A, C1, C2, C3, D1, D2, and D3. GRα-B is produced by the GR gene in the human, monkey, rat, and mouse but not guinea pig due to the second start codon on exon 2 of the guinea pig GR gene containing an isoleucine codon instead of methionine. Alternative start codon positions of human GR are indicated in the Fig 1. In addition, we identified that the guinea pig GR protein is 6 amino acids shorter than the human counterpart. The guinea pig and human GR is composed of 771 and 777 amino acids, respectively. Five residues in the N-terminal domain and one residue in the ligand-binding domain of the human GR are absent in guinea pig GR.

### Effect of gestational age alone on GR Isoforms present in the guinea pig placentae

**Fig 2:**
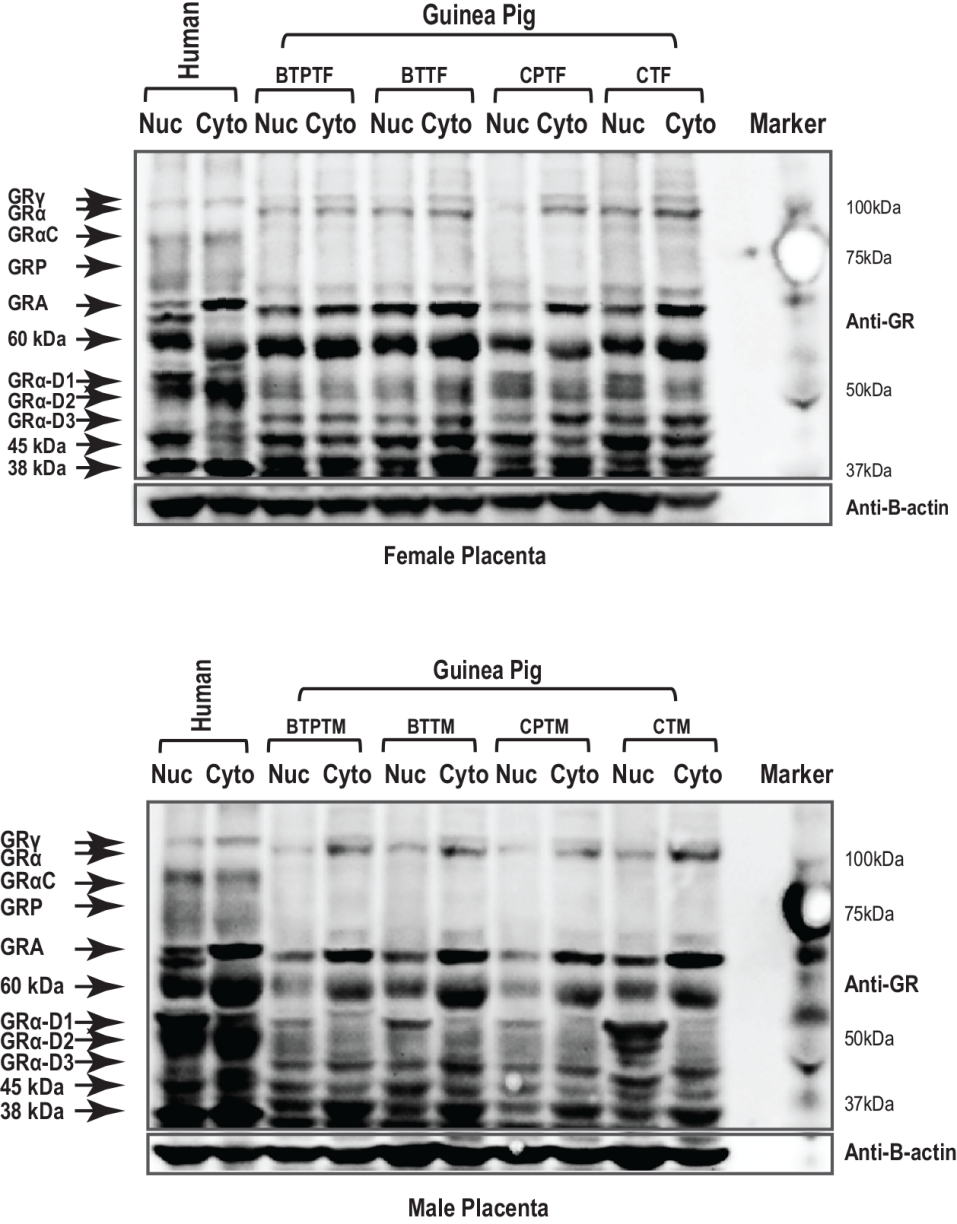
Guinea pig glucocorticoid receptor isoforms in the placentae of pregnancies delivered preterm or at term and in the presence and absence of betamethasone exposure. Glucocorticoid receptor isoforms were measured in cytoplasmic and nuclear fractions of guinea pig placentae. Human placental tissue was used as a positive control and present in lanes 1 and 2 of Fig 2a and 2b. Fig 2a represents female guinea pig placental tissues delivered preterm and exposed to betamethasone (BTPTF) or term female placentae exposed to betamethasone (BTTF) and compared to control preterm females (CPTF) and control term females (CTF). Fig 2b represents placental tissues of male guinea pigs delivered preterm and exposed to betamethasone (BTPTM) or term male placentae exposed to betamethasone (BTTM) and compared to placentae of control preterm males (CPTM) and control term males (CTM).

**Fig 3:**
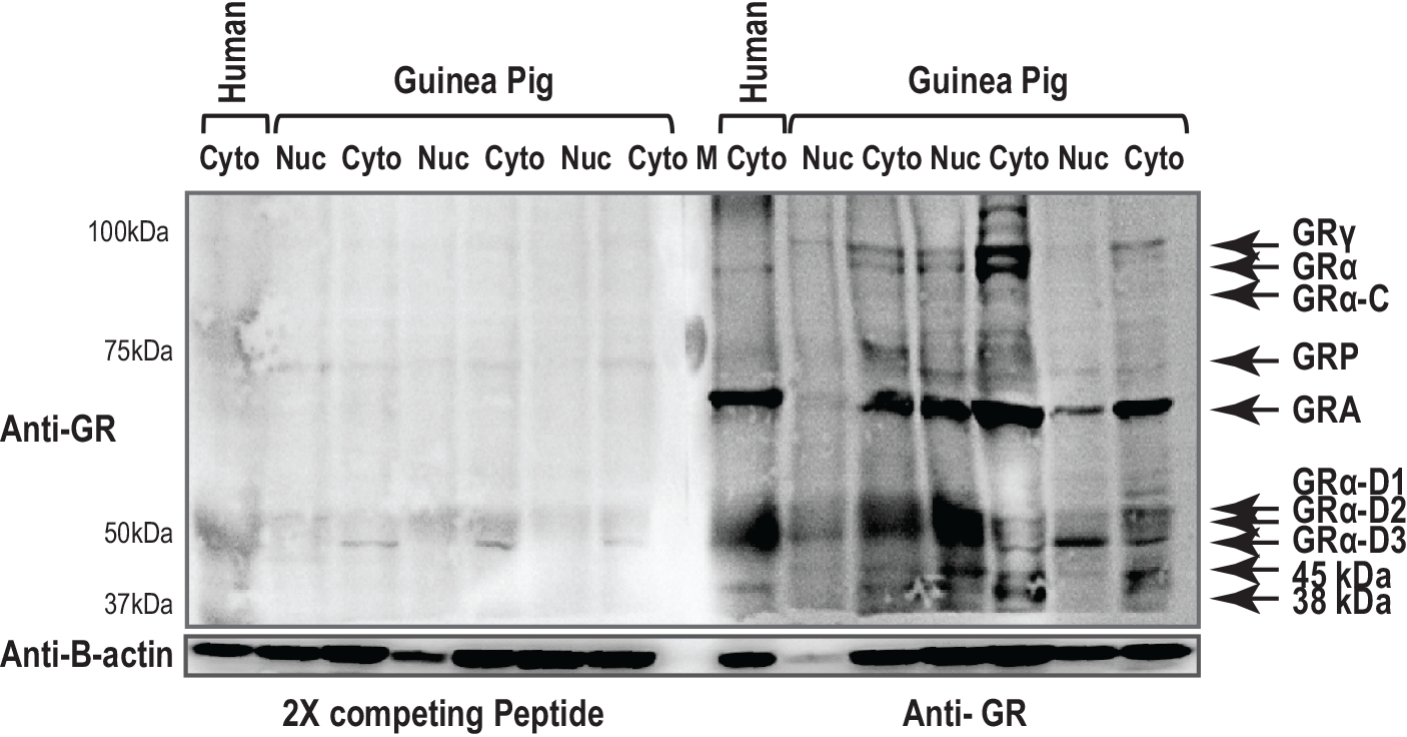
A preabsorption control was conducted to identify nonspecific binding of the antibody. GR antibody was preabsorbed with control peptide prior to Western Blot exposure. The left panel represents binding after GR antibody preabsorption and the right panel is the blot exposed to GR antibody alone. Human placental tissues was included as a positive control.

Total GR antibody (Bethyl Biosciences) identified 10 specific bands in protein nuclear and cytosolic extracts of whole placental tissue derived from term (n = 29) and preterm pregnancies (n = 27). Molecular weights (MW) of 95, 94, 91, 74, 65, 55, 52, 50, 45 and 38 kDa were observed in term placentae (Fig 2). Some of these MWs are equivalent to known isoforms including GRγ (95 kDa), GRα A (94kDa), GRβ (91 kDA), GRP (74kDa), GRA (65 kDa), GRα D1-3 (50–55 kDa). There were unknown proteins detected by the GR antibody including the 45 and 38 kDa proteins. Following pre-absorption of the GR antibody with the control peptide, all MW forms were removed (Fig 3). There were no significant alterations in GRα A with gestational age. Nuclear 38 kDa unknown protein expression was significantly higher in term placentae than preterm placentae (Mann Whitney U test, P = 0.006) (Table 2).

**Table 2:** Placental GR isoform expression in relation to gestational age at delivery, fetal sex and betamethasone exposure.

		Control Preterm			Control Term		Steroid Preterm		Steroid Term		P Value
		Female	Male		Female	Male	Female	Male	Female	Male	
	n	6	7		6	8	6	8	8	7	
**GRγ**	Cytoplasm	0.7	0.07		4.2	2.3	5.23	5.67	0.3	0.8	0.004
	*Range*	*0*.*5–0*.*8*	*0*.*4–2*.*7*		*1*.*9–4*.*9*	*1*.*5–4*.*1*	*3*.*4–7*.*9*	*0*.*9–13*.*0*	*0*.*2–1*.*4*	*0*.*4–1*.*2*	
	Nucleus	0.5	1.2		0.8	1.4	4.29	2.3	0.03	0.6	
	*Range*	*0*.*2–1*.*2*	*0*.*5–2*.*2*		*0–1*.*8*	*0–2*.*8*	*1*.*4–8*.*0*	*0*.*9–7*.*4*	*0*.*3–1*.*5*	*0–1*.*6*	
**GRα A**	Cytoplasm	1.4	0.9		0	11.1	16.6	14.8	1.8	6.1	0.0001
	*Range*	*0*.*7–2*.*0*	*0*.*7–1*.*4*		*0–1*.*7*	*2*.*9–17*.*5*	*10*.*9–20*.*9*	*11*.*1–30*.*4*	*1*.*3–5*.*6*	*1*.*1–6*.*8*	
	Nucleus	1.6	0.7		0.5	7.7	4.82	10.1	1.3	2.4	0.001
	*Range*	*0*.*8–2*.*4*	*0–0*.*8*		*0–1*.*2*	*2*.*3–10*.*6*	*0*.*71–12*.*5*	*4*.*8–19*.*6*	*0*.*9–2*.*1*	*2*.*1–5*.*1*	
**GRβ**	Cytoplasm	0.8	0.4		0	0	2.55	4.2	0.5	2.6	0.01
	*Range*	*0*.*6–1*.*2*	*0–0*.*6*		*0*	*0–3*.*9*	*0*.*3–3*.*57*	*2*.*9–5*.*9*	*0*.*5–1*.*5*	*0*.*3–6*.*0*	
	Nucleus	0.5	0		0	0	2.32	3.5	3	1.8	0.0001
	*Range*	*0–2*.*2*	*0*		*0*	*0*	*1*.*7–2*.*6*	*2*.*4–4*.*7*	*1*.*42–3*.*7*	*1*.*5–3*.*5*	
**GR P**	Cytoplasm	1.5	0.7		2.9	3.5	8.44	9.7	1.4	2.3	
	*Range*	*0*.*6–2*.*3*	*1*.*3–2*.*9*		*1*.*6–4*.*9*	*0–11*.*8*	*5–11*.*2*	*0–22*.*8*	*1*.*2–2*.*9*	*0*.*7–3*.*9*	
	Nucleus	1.4	0		0.8	3.1	0	10.4	1.3	1.4	
	*Range*	*0–2*.*6*	*0–1*.*1*		*0–16*	*0–11*.*2*	*0–0*.*85*	*1*.*0–16*.*6*	*1*.*0–2*.*7*	*0–2*.*0*	
**GR A**	Cytoplasm	2.8	2.9		6.1	22.6	17.4	18.9	2.8	6.1	0.004
	*Range*	*2*.*6–3*.*2*	*2*.*6–4*.*2*		*4*.*3–7*.*2*	*10*.*5–46*.*2*	*10*.*4–22*.*7*	*12*.*9–27*.*0*	*2*.*4–4*.*9*	*1*.*1–10*.*4*	
	Nucleus	2.3	1.5		3.9	13.03	10.1	15.5	2.3	2.8	0.0001
	*Range*	*1*.*6–3*.*0*	*0–1*.*8*		*1*.*5–13*.*8*	*9*.*6–15*.*7*	*5*.*9–11*.*4*	*8*.*4–23*.*3*	*1*.*9–3*.*7*	*2*.*1–5*.*6*	
**GRα D1**	Cytoplasm	0.4	0		3.1	0	5.44	10.9	0.9	2.8	0.02
	*Range*	*0–1*.*1*	*0–0*.*4*		*3*.*1–3*.*4*	*0–4*.*3*	*0–11*.*6*	*4*.*8–18*.*8*	*0*.*4–2*.*2*	*0*.*6–3*.*4*	
	Nucleus	0.5	0		8.4	0	0	8.2	0.7	1.7	0.005
	*Range*	*0–2*.*6*	*0*		*1*.*6–20*.*8*	*0–2*.*2*	*0–4*.*2*	*4*.*7–12*.*7*	*0*.*6–0*.*9*	*0–2*.*6*	
**GRα D2**	Cytoplasm	2.1	0.9		8	6	4.1	16.3	2	5.9	0.01
	*Range*	*0*.*7–3*.*7*	*0*.*4–5*.*9*		*3*.*6–13*.*6*	*1*.*9–6*.*5*	*3*.*3–7*.*6*	*7*.*39–23*.*4*	*1*.*8–2*.*4*	*1*.*2–8*.*2*	
	Nucleus	3.8	1.5		4.7	5.7	7.23	11.4	2.4	4.6	0.02
	*Range*	*1–5*.*1*	*0*.*6–2*.*7*		*3*.*1–24*.*8*	*2*.*8–9*.*4*	*0*.*9–8*.*8*	*7*.*0–17*.*3*	*1*.*7–3*.*7*	*1*.*1–5*.*1*	
**GRα D3**	Cytoplasm	2.1	1.1		0	15.9	9.82	8.1	2	3.7	0.0001
	*Range*	*1*.*7–3*.*0*	*0*.*8–1*.*6*		*0–2*.*5*	*6*.*8–22*.*7*	*6*.*8–10*.*5*	*4*.*6–10*.*8*	*1–4*.*3*	*2–5*.*5*	
	Nucleus	1.7	0		0	12.7	3.4	10.6	1.5	2.5	0.0001
	*Range*	*1*.*6–2*.*1*	*0–1*.*3*		*0*	*3*.*6–21*.*7*	*0*.*63–7*.*23*	*4*.*6–15*.*1*	*1*.*0–3*.*7*	*2–4*.*3*	
**45 kDa**	Cytoplasm	1.2	0.9		0	4	3.85	7	1.3	3.2	
	*Range*	*0–2*.*1*	*0–1*.*0*		*0*	*0–12*.*1*	*0–11*.*9*	*0–14*.*2*	*1*.*1–4*.*8*	*2*.*2–4*.*7*	
	Nucleus	1.4	0		0	4.8	0	0	1.4	2.3	
	*Range*	*0–2*.*3*	*0–0*.*8*		*0*	*0–6*.*9*	*0–5*.*6*	*0–11*.*6*	*1*.*2–1*.*7*	*1*.*6–5*.*3*	
**38 kDa**	Cytoplasm	1.6	1.9		5.5	0	6.96	13.8	1.9	4.5	0.0001
	*Range*	*1*.*4–2*.*3*	*1*.*6–6*.*6*		*3*.*9–8*.*8*	*0*	*6*.*1–11*.*9*	*11–23*.*7*	*1*.*2–3*.*0*	*0*.*7–5*.*7*	
	Nucleus	2.9	1.5		11.9	0	9.73	12.8	2.2	2.6	0.0001
	*Range*	*2*.*4–3*.*3*	*1*.*3–2*.*1*		*3*.*8–23*.*6*	*0*	*5*.*1–18*.*9*	*6*.*5–20*.*6*	*1*.*9–5*.*9*	*2*.*5–5*.*9*	

Data is expressed as median and inter-quartile range.

### Effect of sex alone on placental GR isoform expression

There were very few sex differences in GR isoform expression when examining the entire population (females n = 26, males n = 30). There were no sex differences in the expression of GRα A. Nuclear expression of GRα D3 in males (Mann Whitney U test, P = 0.012) and cytoplasmic expression of 38 kDa protein in females (P = 0.006) were significantly increased at term (Fig 2).

### Effect of betamethasone exposure alone on placental GR isoform expression

Animals were exposed to either vehicle control (n = 27) or betamethasone (n = 29) during pregnancy. Betamethasone treatment was associated with increased cytoplasmic expression of GRα A (Mann Whitney U test P = .001), GRβ (P = 0.001), GRα D 1 (P = 0.014) and D 3 (P = 0.04), 45 kDa (P = 0.02) and 38 kDa (P = 0.003) proteins. In the nucleus there was increased expression of GRα A (P = 0.03), GRβ (P = 0.001), GRα D 3 (P = 0.01), and 38 kDa (P = 0.004) proteins with betamethasone treatment.

### Effect of betamethasone exposure and fetal sex on placental GR isoform expression

When fetal sex was taken into account, cytoplasmic GRα A (Mann Whitney U test, P = 0.001), nuclear GRβ (P = 0.004) and nuclear GRα D3 (P = 0.02) were significantly increased with betamethasone treatment in female placentae. In male placentae, betamethasone treatment was associated with increased nuclear and cytoplasmic GRβ (P = 0.009. P = 0.0001), nuclear and cytoplasmic GRα D 1 (P = 0.002, P = 0.002), nuclear and cytoplasmic GRα D 2 (P = 0.002, P = 0.01) and nuclear and cytoplasmic 38 kDa (P = 0.0001, P = 0.0001) proteins.

### Effect of gestation, betamethasone treatment and sex on GR isoform expression

Placental GRα A was detected in the cytoplasm of all groups but was significantly increased in the cytoplasm and nucleus of preterm males and females exposed to betamethasone and in untreated term males (KW-ANOVA, P = 0.0001, P = 0.001). Cytoplasmic expression of GRγ was decreased in term male and female placentae treated with betamethasone and increased in preterm male and female placentae treated with betamethasone (KW-ANOVA, P = 0.004). Cytoplasmic expression of GRβ was increased with betamethasone exposure in female preterm placentae, preterm and term male placentae (P = 0.01). Nuclear expression of GRβ was increased in all placentae exposed to betamethasone treatment (P = 0.0001). Cytoplasmic and nuclear expression of GR A was increased in male and female placentae exposed to betamethasone and in term untreated male placentae (P = 0.004, P = .0001). Cytoplasmic GRα D1 was increased in male and female preterm placentae exposed to betamethasone (P = 0.02). Nuclear GRα D1 was increased significantly in preterm male placentae exposed to betamethasone and female term control placentae (P = 0.005). Nuclear and cytoplasmic GRα D2 was increased in male preterm placentae when exposed to betamethasone (P = 0.01, P = 0.02). Nuclear and cytoplasmic GRα D3 was increased in untreated, male term placentae (P = 0.0001, P = 0.0001). Nuclear expression of GRα D3 was also increased in male preterm placentae exposed to betamethasone (P = 0.0001). The unknown protein at 38 kDa protein was increased in the nucleus and cytoplasm of preterm placentae exposed to betamethasone and in the nucleus of control term female placentae (P = 0.0001, P = 0.0001).

### Expression patterns and cellular location

**Fig 4:**
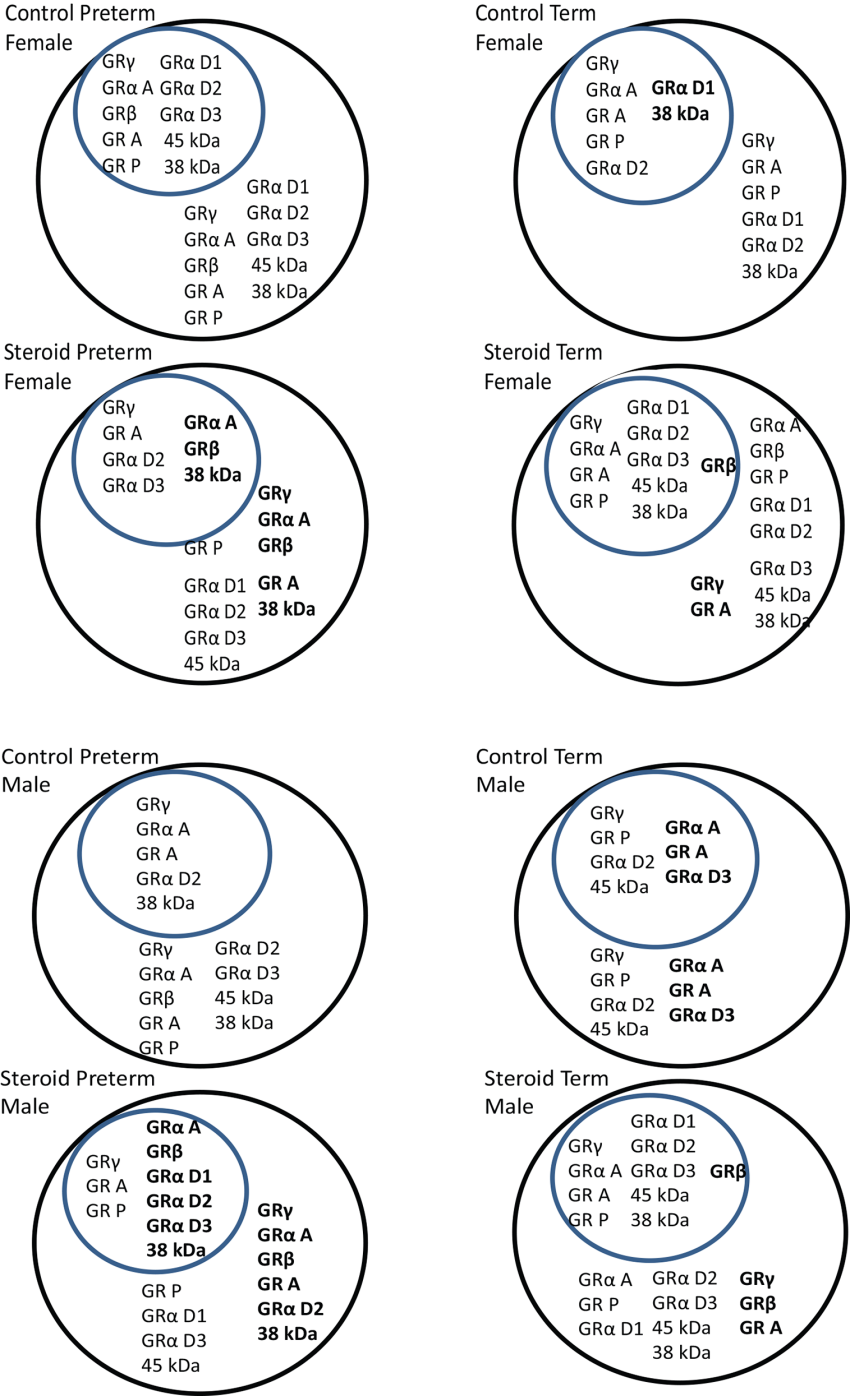
Summary of GR isoform expression in guinea pig placenta in relation to fetal sex, gestational age and betamethasone exposure. The circles represent a cell with the smaller circle being the nucleus and the larger being the cytoplasm. Isoforms that were present under each condition are in small font while isoforms that were significantly altered by treatment, or gestational age are in bold. Fig 4a represents female placentae and 4b represents male placentae. This figure was generated from Table 2 and isoforms were included in the figure if the median value was greater than 0 regardless of whether GR expression was significantly altered or not.

The changing pattern of GR isoform expression with exposure to betamethasone has been summarised in Fig 4. Any GR isoform median value that was greater than 0 in Table 2 was included in this summary.

### GR expression patterns in female placentae

All female placentae expressed GR γ, GRA, GR P, GRα D1, GRα D2 and 38 kDa in the cytoplasm. In the nucleus all female placentae expressed GR γ, GRα A, GR A, GRα D2 and 38 kDa (Fig 4A). The pattern of expression between term and preterm placentae of female guinea pigs was very different as preterm placentae expressed all identified isoforms in the cytoplasm and nucleus. By term, several GR isoforms were no longer expressed including cytoplasmic GRα A, GRβ, GRα D3 and 45 kDa proteins and in the nucleus; GRβ, GRα D3 and 45 kDa were absent. By term nuclear GR A and the 38 kDa protein as well as cytoplasmic GRα D1 were significantly increased relative to untreated preterm placentae. The addition of betamethasone in preterm pregnancies resulted in the absence of GR P, GRα D1 and 45 kDa protein in the nucleus and a significant increase in the expression of nuclear GRα A, GR A and 38 kDa protein. In the cytoplasm of preterm placentae all the isoforms were the same in betamethasone treated pregnancies as those expressed in untreated preterm female placentae except for GR γ, GRα A, GRβ, GRα D1 and 38 kDa which were significantly increased with betamethasone exposure. In term placentae exposed to betamethasone all detected isoforms of GR were present in the nucleus and cytoplasm with a significant increase in the expression of nuclear GRβ and cytoplasmic GRγ.

### GR expression patterns in male placentae

All male placentae expressed GRγ, GRα A, GR A, GR P, GRα D2,GRα D3 and the 45 kDa in the cytoplasm. In the nucleus all male placentae expressed GRγ, GRα A, GR A and GRα D2 (Fig 4B). The pattern of expression between term and preterm placentae of male guinea pigs was associated with a significant increase in the expression of nuclear and cytoplasmic GRα A, GR A and GRα D3 at term. Exposure to betamethasone in preterm placentae resulted in a significant increase in the expression of nuclear GRα A, GRβ, GR A, GRα D1, GRα D2, GRα D3 and the 38 kDa and a significant increase in the expression of GRγ, GRα A, GRβ, GR A, GRα D1, GRα D2, and the 38 kDa in the cytoplasm. Nuclear GRβ was significantly increased in term placentae and all other GR isoforms were present. In the cytoplasm of term placentae exposed to betamethasone, GRβ was significantly increased, GR γ was significantly decreased and all other isoforms were present (Fig 4B).

## Discussion

This study has identified there are 8 known isoforms of the GR in the guinea pig placenta which include 4 translational isoforms; GRα A and GRα D1-3, and 4 splice variants; GRβ, GR P, GR A and GRγ. As predicted by gene matching, GRα B was not detectable in the guinea pig placenta however it was also not identified in the human placenta [21]. GRα C 1–3 was not identified in the guinea pig placenta but was present in the human placenta [21,25]. We have made an assumption that the 95 kDa protein is GR γ but it could also represent a posttranslational modification of a GR isoform or a phosphorylated form of GRα A. There were 2 proteins that were specific for the GR antibody but are currently not associated with any characterised GR isoform. These unknown proteins could be translational isoforms of the splice variants or GR–associated proteins but the fact that these proteins change in association with fetal sex, gestation and betamethasone exposure suggests the latter. The data indicate that the guinea pig may be a suitable model for studying glucocorticoid signalling in the placenta given the GR isoforms are comparable to the human placenta[21].

Significant differences in GR expression were observed when accounting for sex, gestational age and betamethasone exposure and included variations in the expression of several GR isoforms including GRα A and the low transactivational isoforms; GRγ, GRβ, GR A, GRα D1-3. The localisation of the GRα A to the nucleus implies glucocorticoid signalling activity. The data from the control placentae indicated that nuclear GRα A was present but not altered significantly in male placentae in early gestation but increased significantly at term suggesting glucocorticoids may be more bioactive at term in the male. However further studies are required to determine the influence of other GR isoforms on glucocorticoid sensitivity such as GR A and GRα D3 that were significantly increased in male term placentae and may influence the glucocorticoid response to GRα A. Betamethasone treatment significantly increased GRα A in preterm male placentae but had no effect on this isoform at term. Instead, betamethasone treatment at term increased the expression of GR β suggesting the term male placenta attempts to block the effects of glucocorticoid via increased expression of an antagonistic GR isoform. The patterns of expression were different in females relative to males in that control females had low nuclear GRα A expression in both preterm and term placentae. When female preterm placentae were exposed to betamethasone, GRα A was increased in both the cytoplasm and nucleus but at term there was no significant change in expression of GRα A. Similar to male placentae, females also had other low transactivational isoforms present that could influence glucocorticoid bioactivity including GRγ, GRβ and GR A in preterm placentae and GRβ and GR A at term. These particular isoforms have recently been shown to activate and repress a unique set of glucocorticoid regulated genes and it would suggest GRα A-induced pathways are inhibited by the antagonistic effects of GRβ but glucocorticoids may still exert some gene effects via GRα D [17]. The current data raises the possibility of male and female placentae having gestation specific time points of responsivity to glucocorticoid with males being more responsive at term and females being more responsive at mid-gestation.

We have recently examined term and preterm placentae from human pregnancies and identified that there are 8 known isoforms of the glucocorticoid receptor present in the preterm placenta which varied in expression in relation to gestational age at delivery, mode of delivery, fetal sex, betamethasone exposure, fetal size and receptor location in the cytoplasm or nucleus[26]. Similar to the guinea pig there were also several unknown proteins specific for the GR antibody that may be GR isoforms in the human placenta. Furthermore fetal sex was a key contributor to differences in GR expression where female preterm placental GR expression was dependent on birth weight, placental weight and betamethasone exposure but male preterm placental GR expression was unaltered by any of these factors. Interestingly GRα A did not change significantly in the analyses conducted on human preterm placentae and its expression was detectable in 70% of all preterm placentae which was surprising as it was expected to be found in 100% of placentae. Relative to the other GR isoforms, GRα A’s expression was minor constituting less than 2% of all isoforms expressed. The only variation associated with GRα A was that its expression was negatively correlated with preterm female placental weight suggesting cortisol may regulate placental growth via GRα A. Discerning any effects of betamethasone in this cohort of human placentae was difficult due to the variability of gestational age and timing of exposure to betamethasone. Possibly the findings in the guinea pig placenta have more clearly defined the potential impact of betamethasone on the placental GR and point towards its future use for studying the interaction and biological function of multiple GR isoforms.

There are a number of other similarities between the human and guinea pig placenta in relation to the metabolism and response to glucocorticoids that suggest it would be an important animal model for understanding the role of multiple glucocorticoid receptors on placental function and fetal programming. Similar to the human placenta [27,28], the guinea pig placenta metabolises maternal glucocorticoid via 11β hydroxysteroid dehydrogenase type 2 and activity of this enzyme decreases towards term to increase fetal exposure to glucocorticoids [29]. P-glycoprotein, (P-gp), an ATP-dependant efflux pump encoded by the multi-drug resistance 1 (MDR1) gene is present in both the human[30] and guinea pig placenta[31] and involved in the efflux of glucocorticoids from the placental trophoblast back into the maternal circulation. However the guinea pig has been shown to be less sensitive to glucocorticoids than other animal models[32] due to differences in the amino acid sequence within the ligand binding domain of the guinea pig GR when compared to the human GR[33].

Recent studies have identified the transient and premature exposure of the guinea pig fetus to high concentrations of glucocorticoid can have long term effects on hippocampal-hypothalamic-pituitary-adrenal axis function, behaviour and reproductive capacity[34]. These modifications in function are proposed to be induced by global alterations in the methylation status of many glucocorticoid-regulated genes and are inherited by subsequent generations of animals [35]. Epigenetic modifications have also been observed in the placenta but not to the same extent as observed in other fetal organs following betamethasone exposure [35]. In particular the expression of DNA methyl transferases were affected in placenta and varied depending on gestational age and exposure to betamethasone [35]. The physiological relevance to these alterations in the placenta and the contribution to fetal growth and development are yet to be investigated. Most importantly many of these studies have been conducted under the assumption that there may be only a few variants of the GR but the current findings suggest the impact of glucocorticoid exposure on the epigenome may be more complex depending on which GR isoforms are expressed at the time of exposure.

This study has provided evidence that the guinea pig placenta expresses multiple isoforms of the GR in a manner similar to the human placenta. GR isoforms expression varies under a number of conditions depending on gestational age at delivery, placental exposure to betamethasone and fetal sex. These data provide some insight into the effect of in utero exposure to betamethasone on placental tissue and open the way for investigations on downstream signalling pathways.

## Supporting Information

S1 Table Antibodies used for this project. Detailed information on the antibodies and the suppliers
(XLSX)
